# Penile Calciphylaxis Obscured by Phimosis in an End-Stage Renal Disease Patient: A Case Report

**DOI:** 10.7759/cureus.67156

**Published:** 2024-08-18

**Authors:** Chathurangi U Angammana, Hilary Fernando, FHDS Silva

**Affiliations:** 1 Internal Medicine, Colombo South Teaching Hospital, Colombo, LKA; 2 Urology, Teaching Hospital, Ratnapura, Ratnapura, LKA

**Keywords:** hemodialysis, phimosis, penile calciphylaxis, end stage renal disease (esrd), partial penectomy

## Abstract

Penile calciphylaxis is a rare and severe complication primarily observed in patients with end-stage renal disease (ESRD) undergoing dialysis. A 50-year-old man presented with severe penile pain and phimosis. He had a history of hypertension and diabetes mellitus for 10 years, complicated by ESRD and was awaiting a cadaveric kidney transplant. He was on cinacalcet therapy for tertiary hyperparathyroidism. The patient underwent circumcision at which discolouration and necrotic patches involving the glans penis were noted. The histological findings were consistent with calciphylaxis and suppurative inflammation. However, due to persistent severe pain and progressive gangrene, a partial penectomy was performed. This report demonstrates the importance of consideration of calciphylaxis in patients with ESRD when presenting with penile pain, even phimosis.

## Introduction

Penile calciphylaxis, also known as calcific uraemic arteriolopathy, is a rare but serious clinical entity caused by calcification of arterioles of the dermis and adipose tissue [1]. It has gained much attention due to its high mortality and morbidity, especially among end-stage renal disease (ESRD) patients on haemodialysis. Progressive calcification of small to medium-sized blood vessels within the subcutaneous tissue of the penile region leads to ischaemia, tissue necrosis, and often excruciating pain as characteristic features [2]. Though, calciphylaxis mainly affects the skin, its manifestation in the penile region is exceptionally uncommon and can pose alarming challenges in diagnosis, management, and treatment [3]. Presentation of penile calciphylaxis with phimosis in the background of penile pain is a rare occurrence [4]. This case report highlights such a concealed clinical presentation along with its diagnostic approach, and multidisciplinary management in a 50-year-old male patient with ESRD undergoing dialysis, underscoring the complexity of this condition and the need for heightened clinical awareness.

## Case presentation

**Figure 1:**
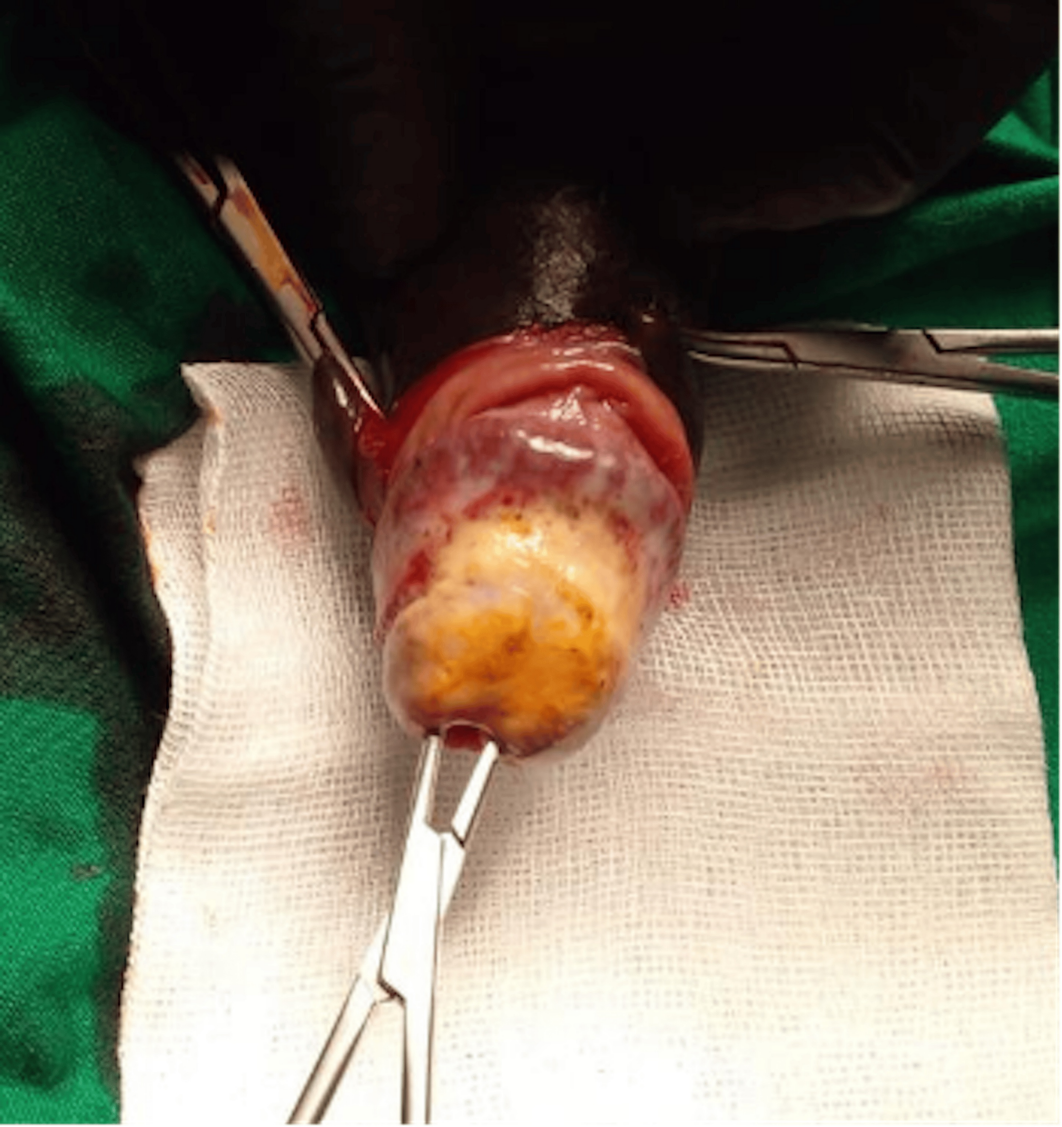
Discolouration of the glans penis at the time of circumcision suggestive of calciphylaxis

A 50-year-old male with a 10-year history of hypertension and diabetes mellitus, complicated by ESRD, presented with severe penile pain of two weeks duration. He was on twice-weekly haemodialysis and was awaiting kidney transplantation. There was swelling and induration on the glans penis with non-retractile foreskin suggestive of phimosis. A penile dorsal slit converting to a circumcision demonstrated discolouration of the glans penis (Figure 1). Calciphylaxis of the glans penis was considered at this stage and an excision biopsy of the lesion was performed.

The patient did not have a history of limb claudication suggestive of peripheral artery disease. He had erectile dysfunction for two years attributed to diabetes mellitus and advanced renal disease. He did not have recurrent oral ulcer, alopecia, vasculitic type of skin rashes, or Raynaud’s phenomenon suggestive of connective tissue disease. There was no history of application of medication to the penis. He did not have a history of sexual promiscuity and had no history of consumption of alcohol or smoking.

The patient was afebrile, pale, and no evidence of lymphadenopathy was found. He had a functioning arteriovenous (AV) fistula on the right upper limb. A cardiovascular examination revealed blood pressure of 140/100 mmHg and a pulse rate of 88 /minute. All peripheral pulses were felt. Respiratory, neurological, and abdominal examinations were unremarkable. Investigations of the patient are given in Table 1.

**Table 1:** Laboratory investigations CRP: C-reactive protein; ESR: erythrocyte sedimentation rate; [Ca2+×PO43-]: calcium phosphate product; PTH: parathyroid hormone; ALP: alkaline phosphatase; SGOP: serum glutamic oxaloacetic transaminase; SGPT: serum glutamate pyruvate transaminase, HIV: human immunodeficiency virus; ANA: antinuclear antibody; HSV: herpes simplex virus

Investigations	Patient’s values	Normal Values
White blood cells	7 x 10^9^/L (Neutrophils 62%, Lymphocytes 30%)	4-10 x 10^9^ /L
Haemoglobin	9.8 g/dl	12-16 g/dL
Platelets	230 x 10^9^/L	150-400 x 10^9^ /L
CRP	54 mg/L	<6
ESR	66 mm 1^st^ hour	
HbA1c	7.8%	<6.5%
Serum Creatinine	9.6 mg/dl	0.7-1.3 mg/dl
Serum albumin	3.0 g/dl	3.5-5.5 g/dl
Ionized Ca^2+^	4.6 mg/dl	4.5-5.3 mg/dL
Corrected Calcium	10.1 mg/dl	8.5-10.2 mg/dl
Serum PO_4_^3-^	9.7 mg/dl	2.8-4.5 mg/dl
[Ca^2+^×PO_4_^3-^]	97.97 mg^2^/dl^2^	>70 mg^2^/dl^2^ associated with metastatic calcifications
PTH level	131 pg/ml	10-55 pg/ml
ALP	167.2 IU/l	30-120 IU/l
SGOT	15.1 U/L	<50
SGPT	11.7 U/L	<50
Urine full report	Pus cells - 3 to 4 Red blood cells - nil Protein - +	
Blood culture	No growth	
Urine culture	No growth	
HIV I and II ab	Negative	
Urethral swab for gonococcal and chlamydial disease	Negative	
HSV serology	Negative	
ANA	Negative	

**Figure 2:**
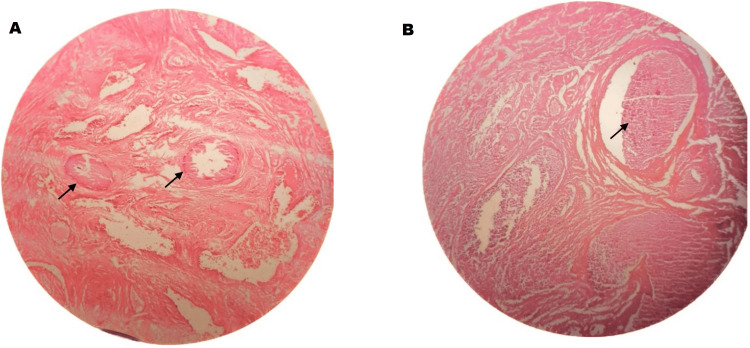
Photomicrographs (A: Right side, B: Left side) of excision biopsy of glans penis with haematoxylin and eosin stain, showing extensive suppuration, tissue necrosis, with focal calcification

Ultrasonography of the neck did not show evidence of parathyroid enlargement. The histological sections revealed large areas of tissue necrosis involving the soft tissue of the glans penis. Scattered deposits of dystrophic calcification were noted supporting the diagnosis of penile calciphylaxis. There were no features of dysplasia or malignancy (Figure 2). The histology of the foreskin was normal. 

The patient was commenced on cinacalcet 30 mg initially and was increased to 60 mg twice a day. The haemodialysis plan was escalated to three times a week. Subsequently, the discolouration of the penis progressed to the development of dry to wet progression of gangrene of the glans penis although notably in the absence of systemic signs such as fever or concurrent necrotic or ulcerative lesions elsewhere on the body. Despite these further non-operative modalities and meticulous wound care, there was a persistence of incapacitating pain and progression of the dry gangrene. Consequently, a multidisciplinary consensus led to the decision to proceed with partial penectomy as a therapeutic intervention. The symptoms related to penile discomfort subsided.

**Figure 3:**
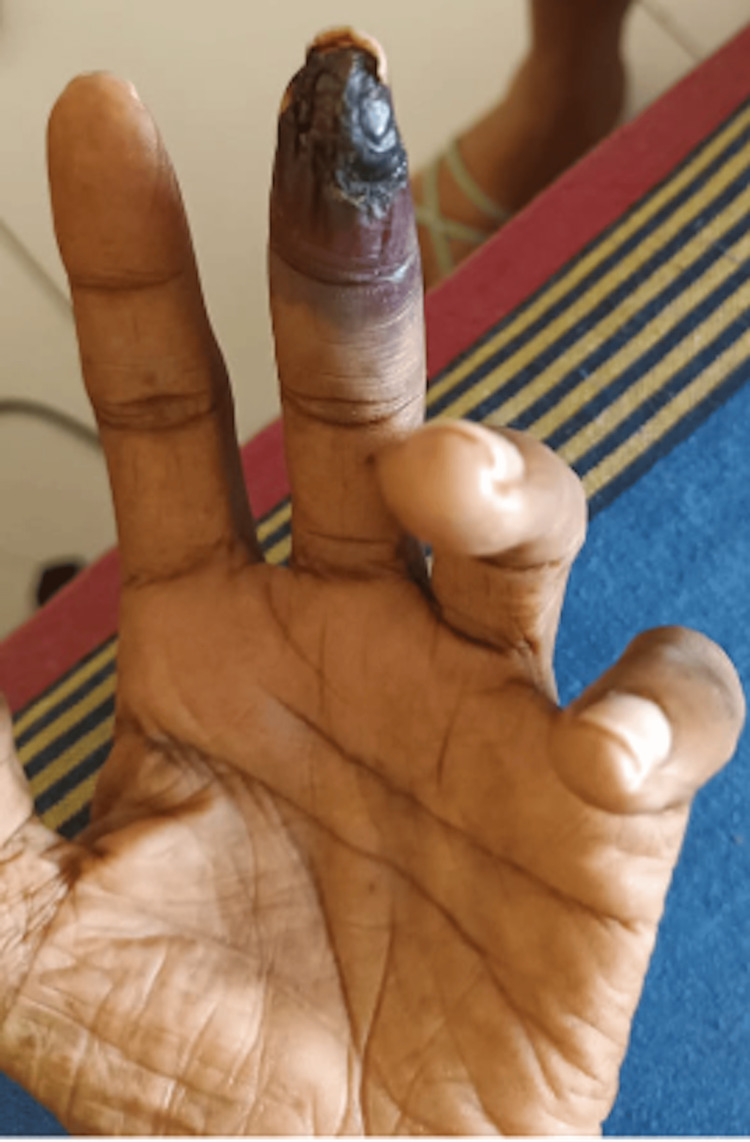
Subsequent calciphylaxis developing in the third finger resulting in gangrene

However, six months later, he started developing similar calciphylaxis of the left middle finger for which he refused surgical options including wound debridement. The finger autoamputated in three weeks (Figure 3). He succumbed at the same time to an acute ST elevated myocardial infarction.

## Discussion

Penile calciphylaxis is a catastrophic uncommon condition characterized by the calcification of small calibre blood vessels-media of arterioles and capillaries in the penile region, leading to tissue necrosis and often excruciating pain [5]. While calciphylaxis predominantly affects individuals with ESRD, its manifestation in the penile tissue is exceptionally rare and presents unique diagnostic and therapeutic dilemmas for clinicians [6]. The clinical presentation was obscured with phimosis, impeding the early diagnosis before circumcision. The literature is limited to this phenomenon of non-retractile foreskin presenting as penile calciphylaxis. Thus we emphasise the importance of considering this entity in those with ESRD to prevent advanced mutilating disease.

As seen with the patient, calciphylaxis primarily affects individuals with advanced kidney disease, particularly those undergoing haemodialysis. The exact prevalence of penile calciphylaxis remains unknown due to its rarity, but it is estimated to occur in less than 1% of patients with ESRD [5,7]. However, calciphylaxis has occasionally been described to occur before dialysis [8]. The pathogenesis of penile calciphylaxis is multifactorial, involving disturbances in mineral and bone metabolism, vascular calcification, endothelial dysfunction, and hyperparathyroidism [9]. The pathogenic mechanism of calciphylaxis is based on the hypothesis of metastatic calcification although the exact mechanism remains an enigma [10]. Calcium deposition is observed in the media of small vessels accompanied by intimal thickening and luminal narrowing resulting in distal ischaemia.

Deranged calcium-phosphate metabolism and hyperparathyroidism result in a high [Ca2+×PO43-] product. A [Ca2+×PO43-] more than 70 mg^2^/dl^2^ is associated with metastatic calcification. Approximately 80% of patients with calciphylaxis were found to have secondary or tertiary hyperparathyroidism as a result of renal failure [11]. Other than ESRD and long duration of haemodialysis and diabetes mellitus, the risk of calciphylaxis increases with hypoalbuminaemia, hyperphosphataemia, warfarin intake, and autoimmune disease.

The uraemic state has also been involved in the pathogenesis by causing dysfunction in calcification inhibitors [12]. A recent study comparing α2-Heremans-Schmid glycoprotein/ fetuin-A (AHSG) level, a major systemic calcification inhibitor, among healthy individuals and haemodialysis patients has shown a significant reduction among haemodialysis patients [13].

Patients with penile calciphylaxis typically present with intense penile pain, discolouration, and the development of non-healing ulcers or necrotic lesions in the penile region. The pain is often disproportionate to physical findings and can significantly impair the quality of life. Differential diagnoses include infections, dermatological conditions, and penile malignancies, necessitating a comprehensive evaluation to establish the correct diagnosis [14].

The diagnosis of penile calciphylaxis is based on clinical findings, laboratory tests, and imaging studies. Laboratory investigations commonly reveal abnormal calcium-phosphate metabolism, including elevated serum calcium, phosphorus, and parathyroid hormone levels [15]. Studies have found that, among patients with calciphylaxis, 68% had hyperphosphatemia, 20% hypercalcemia, and 33% had elevated [Ca2+×PO43-] product greater than 70 [15]. Our patient had evidence of high normal levels of calcium with significant hypercalcaemia accompanied by raised serum parathyroid hormone levels defining tertiary hyperparathyroidism with a [Ca2+×PO43-] of 141.77 mg2/dl2. Imaging modalities such as ultrasound and computed tomography may demonstrate vascular calcifications and aid in assessing the extent of tissue involvement [16-18]. However, we were unable to arrange these radiological investigations due to limitations in logistics.

The management of penile calciphylaxis is multifaceted and often requires a multidisciplinary approach involving nephrologists, urologists, radiologists, wound care specialists, and dermatologists [9]. Conservative measures focus on optimizing renal replacement therapy, controlling mineral imbalances, and providing meticulous wound care. Pharmacological interventions, including sodium thiosulfate and cinacalcet, have shown promising results in select cases [1,19]. Medical parathyroidectomy is attempted by employing cinacalcet, a calcimimetic although there is ambiguous evidence between metanalysis and systemic reviews [1]. Sodium thiosulphate due to its calcium chelator, antioxidative and vasodilatory properties, is used as a medical treatment [6].

Surgical parathyroidectomy is used in those who are poorly responsive to medical management [1]. However, our patient was not fit to undergo general anaesthesia. Refractory cases may necessitate surgical interventions such as partial penectomy to alleviate pain and prevent further tissue necrosis as in this patient [20]. Hyperbaric oxygen therapy has also shown some promising results in treatment [6].

Despite advances in understanding and management, penile calciphylaxis remains a challenging condition with limited therapeutic options. The rarity of the disease poses significant challenges in conducting large-scale clinical trials and developing evidence-based guidelines. Further research is warranted to elucidate the pathophysiology of penile calciphylaxis, identify novel diagnostic biomarkers, and explore targeted therapeutic interventions. Additionally, raising awareness among healthcare providers about the clinical presentation and management strategies for penile calciphylaxis is imperative to improve patient outcomes.

## Conclusions

Penile calciphylaxis is a rare but devastating complication of advanced kidney disease, with a poor prognosis. This can present with an obscurity with pain in the penis accompanied by phimosis. Despite its rarity, the disease warrants prompt recognition and multidisciplinary management to improve patient outcomes and quality of life. Attention should be on preventing this condition by controlling predisposing conditions, as therapeutic actions are limited so far. 
